# Life’s homochirality: Across a prebiotic network

**DOI:** 10.1073/pnas.2505126122

**Published:** 2025-08-19

**Authors:** S. Furkan Ozturk, Dimitar D. Sasselov

**Affiliations:** ^a^Harvard-Smithsonian Center for Astrophysics, Cambridge, MA 02138; ^b^King’s College, Cambridge CB2 1ST, United Kingdom

**Keywords:** homochirality, origin of life, RNA

## Abstract

For centuries, scientists have been puzzled by the mystery of life’s biomolecular homochirality—the single-handedness of biological compounds. Sugars and nucleic acids are right-handed, while amino acids are left-handed in biological systems. Likewise, certain metabolites are homochiral, though their handedness varies. However, efforts to address the homochirality problem have often focused on a single compound, a single molecular class, or invoke an extraterrestrial origin. Here, we emphasize the importance of achieving homochirality across an entire prebiotic chemical network and explore a terrestrial pathway for its emergence. This pathway is supported by recent experimental results from several independent studies, as well as analyses of pristine asteroid materials. Our analysis identifies the genome as a key site for achieving network-scale homochirality on early Earth and addresses the opposite handedness of *D*-nucleic acids and *L*-peptides in biology through nonenzymatic, stereoselective coded peptide synthesis.

Life is homochiral: its molecular building blocks—nucleosides and amino acids—appear in only one of two possible mirror-imaged forms, called enantiomers. Yet, due to their energetic and thermodynamic equivalence, chemistry typically produces and sustains such chiral molecules in a racemic (50:50) mixture. The mystery of life’s homochirality was first noted by Louis Pasteur (1) and further explored by Emil Fischer, whose nomenclature we still use today: *D* (dextro) for life’s sugar enantiomers and *L* (levo) for the amino acids (2). Since Fischer’s early insights into the stereoselective nature of enzymatic reactions, we now understand why life relies on maintaining homochiral purity to survive despite the natural tendency toward racemization. Moreover, a very recent careful analysis of pristine amino acid samples from Bennu and Ryugu “challenges the hypothesis that the emergence of left-handed protein-based life on Earth was influenced by an early Solar System bias toward L-amino acids (3).” Therefore, both chemical reasoning and experimental evidence (4) suggest that homochirality must have originated on Earth, during prebiotic chemistry, enabling the efficient and selective synthesis of functional polymers like nucleic acids—which were likely essential for life’s emergence.

However, the mechanism by which homochirality was established remains unresolved. Numerous scenarios have been proposed (5), too many to detail here. Many suggest a source of initial symmetry breaking—a chiral agent, capable of inducing an enantiomeric excess (e.e.) of *D* or *L* forms. Several proposals add the necessary step of amplification—how this initial e.e. might be amplified to achieve full homochirality (e.e. = 100%). Only recently, however, has a third critical ingredient been highlighted—the need for the initial chiral information to propagate with high fidelity across the entire prebiotic synthetic network (6). A growing body of experimental evidence from the past few years suggests that we may be on the cusp of uncovering how life’s homochirality emerged on early Earth—across a prebiotic network.

## Propagation of Chiral Information from Nucleic Acids

The necessity of this third ingredient is best explained with an example. Consider tartrate—a key metabolite precursor whose crystals were identified and separated by Pasteur in his 1848 experiments. Tartrate crystallizes as a conglomerate, with each enantiomer forming its own enantiopure crystal, allowing for its chiral resolution. This initial chiral information can then be transferred through reduction to malate, a central metabolite in the Krebs cycle. However, this transfer stops there, with no possibility of propagation to other members of the cycle, and more critically, to any amino acid or sugar. The issue arises because malate is flanked by two achiral metabolites-oxaloacetate and fumarate. Similarly, metabolic synthesis of homochiral sugars from the achiral precursor pyruvate is problematic in the absence of homochiral enzymes. In fact, any attempt to propagate chirality through metabolic cycles faces this problem: Without enzymes, metabolic cycles act as “chirality grinders,” destroying chiral information.

Amino acids also seem to present problems for chiral propagation, although to a lesser extent than metabolites. Their idiosyncratic synthesis pathways and varying chemical and physical properties make resolving each amino acid individually a tedious task. Additionally, their inconsistent interactions with chiral physical fields (nine out of the nineteen *L*-amino acids, based on Fischer’s convention, are dextrorotatory when measured at the sodium D-line) may result in the selection of amino acids with opposite handedness under the same chiral influence. This constraint suggests that the selection of left-handed amino acids happened through chemical means via stereoselective interactions if chiral information can be uniformly transferred to all amino acids from another molecular class.

Alternatively, catalytic ligation reactions in a mixture of amino acids can amplify a small enantiomeric bias and lead to the formation of homochiral dipeptide precursors (7), offering insights into how such ligation reactions may have amplified homochirality in early, noncoded peptides. However, as Higgs and Blackmond also acknowledge, “addressing the issue of homochirality in the genetic polymers of RNA and DNA remains key to the origin of life” (8).

Moreover, the historical interest in amino acids as the source of life’s homochirality now seems less compelling in light of recent analyses of samples from Bennu and Ryugu (3). These studies indicate that earlier reports of enantioenrichment in meteoritic amino acids were likely due to terrestrial contamination rather than evidence of an extraterrestrial chiral bias. In retrospect, the absence of enantiomeric bias in the original Murchison analysis (9), as well as in the promptly recovered-and thus minimally exposed-Winchcombe meteorite, which happened to fall on a driveway (10), further supports this conclusion. These landmark findings not only challenge the long-standing focus on amino acids but also bring the problem-quite literally back down to Earth.

**Fig. 1.**
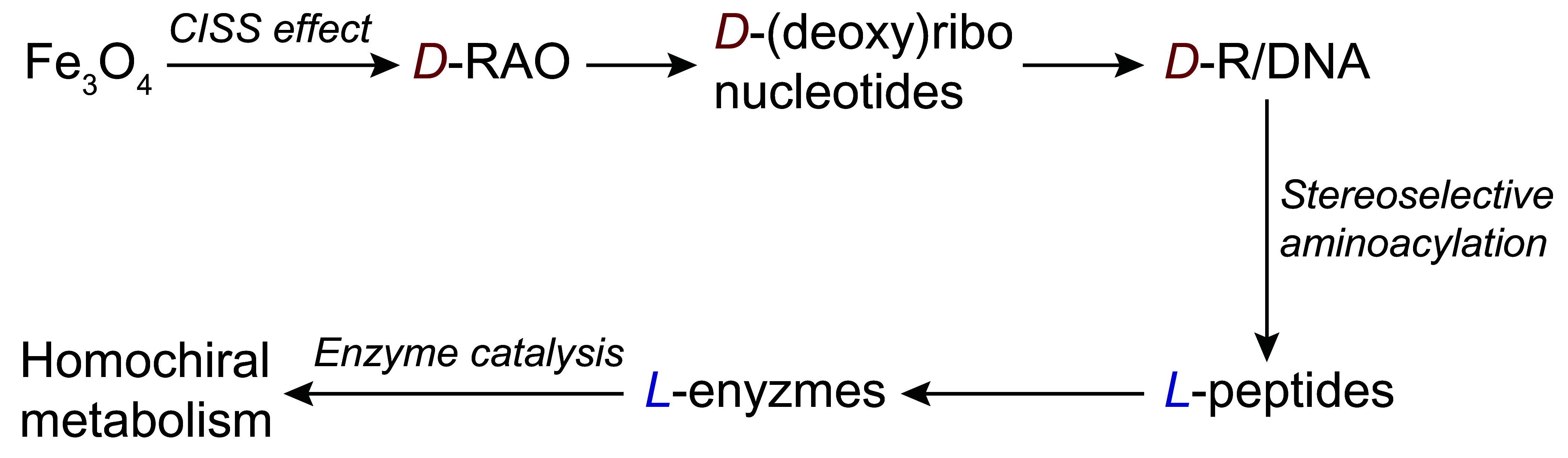
The propagation of chiral information: from a single compound-a shared precursor of RNA and DNA, ribose-aminooxazoline (RAO), on magnetite (Fe_3_O_4_)—all the way to a homochiral metabolism. In this model, chiral symmetry breaking is initiated on magnetized magnetite surfaces by the chiral-induced spin selectivity (CISS) effect, inducing an enantiomeric excess. This excess is subsequently amplified to full homochirality through the conglomerate crystallization behavior of RAO, a nucleotide precursor, which is then transferred to the early genome. From there, chiral information propagates efficiently to peptides through stereoselective coded peptide synthesis and then to metabolites via enzyme catalysis. This approach simplifies the massive problem of life’s biomolecular homochirality to the formation of a single common nucleic acid precursor, RAO, in a homochiral form, enabling network-wide homochirality to be established and maintained.

Recognizing these issues, last year Ozturk, Sasselov, and Sutherland (6) proposed a “central dogma”-like unidirectional flow of chiral information from nucleic acids to peptides, ultimately leading to metabolites by enzyme catalysis (Fig. 1). This chiral information transfer framework is analogous to Francis Crick’s central dogma of genetic information transfer in biology, from nucleic acids to proteins (11).

Their genome-centric approach begins with the origin of homochirality in a shared genomic precursor, ribose-aminooxazoline (RAO). This is achieved by RAO’s enantioselective crystallization on a magnetized magnetite surface due to a recently discovered phenomenon: chirality-induced spin selectivity, or CISS. The CISS effect establishes a strong coupling between electron spin and molecular chirality, enabling achiral magnetized surfaces to function as chiral agents (12). Ozturk et al. demonstrated that magnetized magnetite, a common mineral on early Earth, can lead to enantiopure RAO (13), with which homochirality can be achieved in RNA and then propagated to peptides.

Their model of chiral information transfer builds on pioneering nonenzymatic aminoacylation experiments with *D*-tRNA analogs (14) and recent findings that showed a 10:1 stereoselective preference for *L*-alanine peptides over *D*-alanine peptides (15). The stereoselectivity observed in coded peptide synthesis could enable the transfer of chiral information from *D*-nucleic acids to *L*-peptides, potentially reducing the colossal problem of life’s homochirality to obtaining a single common nucleic acid precursor in its homochiral form. This model of chiral information transfer avoids assumptions about specific chiral agents, amplification methods, or the synthesis pathways of chiral biomolecules. Instead, it focuses on the direction of chiral information transfer and the optimal molecular class for establishing homochirality.

Recent experimental findings from multiple labs have provided evidence supporting the transfer of chiral information from nucleic acids to peptides. Roberts et al. observed stereoselectivity in the nonenzymatic synthesis of aminoacyl-RNAs via phosphoramidates for valine, leucine, and serine, favoring *L*-amino acids on *D*-tRNA (16). Similarly, Radakovic et al. examined the nonenzymatic aminoacylation of *D*-RNA sequences and reported moderate stereoselectivity in the formation of amino acid-bridged stem-loop RNAs with alanine and phenylalanine, in favor of *L*-amino acids (17). Most recently, Kim et al. explored the stereoselectivity of aminoacyl-RNA loop-closing ligation (18). Their study not only revealed a strong preference for *L*-amino acids—specifically alanine, leucine, lysine, and proline—with stereoselectivities reaching up to 200-fold but also demonstrated that a mirror-imaged RNA system favors the opposite-handed amino acids (18). Thus, the findings of Kim et al. provide compelling and comprehensive evidence that RNA chirality is indeed responsible for the observed stereoselectivity in the nonenzymatic formation of aminoacyl-RNAs, strengthening our model’s emphasis on nucleic acids’ centrality for the emergence of network-wide homochirality.

In alternative system, Węgrzyn et al. showed a preference for the coupling of homo-*L*-peptides of various amino acids on right-handed RNA strands (19). However, in their previous studies of an RNA-peptide world, which serves as the foundation for the current work, the authors emphasize that the “obtained RNA-peptide chimeras are not considered to be the direct precursors of the modern ribosome” (20). As such, while these systems exhibit stereoselectivity, they cannot continuously evolve into the molecular machinery observed in contemporary biology, violating the principle of continuity (21). Seemingly contradictory findings by Kenchel et al. showed no consistent stereoselectivity in a ribozyme-catalyzed aminoacylation experiment (22). However, it is crucial to note that this enzymatic process utilized biotin, a prebiotically implausible and chiral substrate. Until the origin of stereoselectivity in this system is clarified and a plausible substrate is used, these results remain less applicable to the study of prebiotic chiral information transfer.

Although the experimental methods and origins of stereoselectivity differed, researchers studying nonenzymatic aminoacylation and peptide-bond formation on homochiral tRNA analogs consistently observed a stereoselective bias in the formation of *L*-peptides from *D*-RNA and vice versa. As suggested in ref. 6, these findings highlight the critical role of RNA-templated peptide bond formation in propagating homochirality from RNA to peptides as a general scheme, regardless of the specific amino acids involved. Collectively, these recent results, alongside previous work, provide a clear framework for understanding the emergence of biomolecular homochirality across multiple molecular classes once it is established in the protogenome.

In our original model we considered enzyme catalysis as a way to propagate chiral information from coded peptides to metabolites, considering an eventual and continuous convergence to modern biology, where stereoselectivity is controlled by proteins. However, homochiral metabolism can also emerge without relying on protein catalysis, instead being facilitated by ribozyme catalysis directly. Considering the substantial evidence that *D*-RNA is naturally inclined to function with *L*-amino acids, it is plausible to envision the propagation of homochirality through an RNA-catalyzed metabolism. In such a system, *D*-ribozymes would catalyze metabolic reactions, including the synthesis of homochiral *L*-amino acids. Once such a system evolves, the subsequent development of translation and the synthesis of homo-*L*-proteins would become inevitable. This pathway presents a complementary paradigm for the propagation of chiral information from nucleic acids to a homochiral network.

Moreover, our analysis indicates that homochirality cannot emerge and be propagated from metabolism alone. Mayer and Moran acknowledge this limitation and propose a pathway for propagating homochirality through the stereoselective nonenzymatic reduction of keto acids by nicotinamide adenine dinucleotide hydride (NADH), whose chirality stems from its two nucleoside units (23). Although the proposed stereoselectivity has not been verified experimentally, they note that “under prebiotic conditions, nucleosides like the adenosine fragment in NADH can be synthesized from ribo-aminooxazolines, which have recently been shown to be obtainable in homochiral form,” suggesting that homochirality should propagate from nucleotide precursors.

## From Magnetic Minerals to a Homochiral Network

How could such a homochiral network emerge in nature? Previous studies by Ozturk et al. suggest that the initial chiral symmetry breaking could have a magnetic origin, through the CISS effect (13). This mechanism provides a planetary hemisphere-wide uniformity for the chiral agent (magnetic mineral surfaces magnetized under the geomagnetic field), which is stable much longer than chemical timescales relevant for the origin of life. Furthermore, the enantiomeric excess induced by magnetized magnetite (Fe3O4) surfaces could be amplified to homochirality due to the rare conglomerate crystallization of RAO. RAO then produces pyrimidine ribonucleotides and purine deoxyribonucleotides (24), which could form an early genome via Watson–Crick base pairing (Fig. 1). Therefore, a functioning homochiral genome can be produced from a central precursor with essential physical properties, RAO, which can be made homochiral through a persistent physical mechanism on magnetic mineral surfaces, starting from fully racemic materials.

Although this symmetry-breaking and amplification process on RAO relies on more assumptions regarding the chiral agent, mechanism, and prebiotic chemistry than the proposed chiral information transfer model, it remains feasible across a variety of early Earth environments. Magnetic minerals, such as magnetite and greigite, which carry remanent magnetization from the planetary magnetic field, are found abundantly. Surface lakes with wet-dry cycling can facilitate the concentration and subsequent crystallization of materials. The main requirement is to execute this symmetry-breaking process for an RNA precursor-or, more generally, a genomic precursor-so that the resulting homochirality can be effectively propagated, leading to a homochiral network.

## Outlook

The origin of life’s homochirality is a network-scale problem; thus, focusing on the homochirality of a single compound or molecular class falls short of providing a comprehensive understanding. Effective propagation of homochirality imposes a stringent constraint on potential scenarios for the origin of life. Without specific assumptions about prebiotic chemical synthesis or the initial symmetry-breaking mechanism, the genome emerges as a more plausible starting point for homochirality than peptides or metabolites. Homochirality, once established in the genome, can propagate to peptides via coded peptide synthesis-as recent work supports-and subsequently to metabolites through enzyme catalysis, indicating a unidirectional transfer of chiral information (Fig. 1). This directional flow parallels the sequential flow of information described by the central dogma of molecular biology. Just like the sequential information in Crick’s central dogma, in our model, chiral information flows from nucleic acids to peptides-not backward.

Based on this genome-centered origin of homochirality, we propose that the homochirality problem can be addressed most effectively within a genome-centric framework for life’s origins, recognizing homochirality as an essential prerequisite for the emergence of life. Furthermore, our previous experimental work shows that homochirality can indeed be achieved in RNA from completely racemic starting materials, highlighting magnetic mineral surfaces as plausible agents for chiral symmetry breaking due to the CISS effect. The abundance of magnetic minerals, such as magnetite, supports the prebiotic plausibility of this mechanism, which could reliably take place across various environments on early Earth and other planets.

## Data Availability

There are no data underlying this work.
